# Intraosseous Meningioma Along the Left Petrous Bone: A Rare Cause of Trigeminal Neuralgia

**DOI:** 10.7759/cureus.32414

**Published:** 2022-12-11

**Authors:** Phoebe Lim, Akram M Eraky, Dylan Coss, Nathan Zwagerman

**Affiliations:** 1 Neurosurgery Department, Medical College of Wisconsin, Milwaukee, USA; 2 Pathology Department, Medical College of Wisconsin, Milwaukee, USA

**Keywords:** skull base surgery, bony skull tumors, trigeminal nerve decompression, middle fossa craniotomy, secondary trigeminal neuralgia, neurological tumors, petrous bone, trigeminal neuralgia, intraosseous tumor, meningioma

## Abstract

Trigeminal neuralgia (TN) presents with symptoms of intense recurrent shock-like brief pain localized to specific areas of the face innervated by the fifth cranial nerve. The pathology of trigeminal neuralgia is attributed to the fifth cranial nerve compression or demyelination. Most cases of this diagnosis are not due to bony structures, making this case an uncommon presentation of trigeminal neuralgia. Herein, we present a case of trigeminal neuralgia due to an intraosseous meningioma that formed along the left petrous bone, resulting in trigeminal nerve compression. On head computed tomography (CT), osteomatous growths along the left petrous bone were noticed compressing the trigeminal nerve. After trigeminal nerve decompression and drilling out the protruding part of the petrous bone through middle cranial fossa craniotomy, the patient’s symptoms were completely improved postoperatively and at the two-month follow-up. To our knowledge, there are only four reported cases of trigeminal neuralgia caused by petrous bone compression in the literature. We emphasize the significance of considering petrous bone lesions as a cause of trigeminal neuralgia.

## Introduction

Trigeminal neuralgia (TN) is characterized by recurrent shock-like brief pain restricted to unilateral trigeminal divisions [1,2]. TN occurs due to many causes related to nerve degeneration, such as vascular compression, mass compression, and multiple sclerosis [1-7]. Of interest, only a few cases of trigeminal nerve compression by bony structures have been reported [8-11]. This specific case presents a patient diagnosed with TN due to an intraosseous meningioma that formed along the left petrous bone, leading to trigeminal nerve compression. To our knowledge, only four cases of trigeminal neuralgia that are caused by petrous bone growths are reported in the literature [8-11].

## Case presentation

A 32-year-old female arrived at our hospital due to increasing facial pain over the left back of her jaw that extended halfway down the mandible with recurrent episodes of shock-like pain, which was grade 4 on the Barrow Neurological Institute (BNI) pain scale. At the time, the patient stated that there had been more facial pain, increasing in severity with each passing day. Most of the pain was left-sided, with occasional right-sided pain. She also experienced temporal pain. Her past medical history includes difficulties with muscle pain along her jaw and cramps in the masseteric region. She also received multiple Botox injections to the masseters, temporalis, and lateral pterygoids. For a while, headaches and pain significantly improved temporarily. However, her symptoms were not fully resolved. Other forms of treatment did not provide long-term relief and included methods of dry needling, physical therapy, medication, and chiropractic adjustment. The patient has a past medical history of fibromyalgia and postural orthostatic tachycardia syndrome.

**Figure 1:**
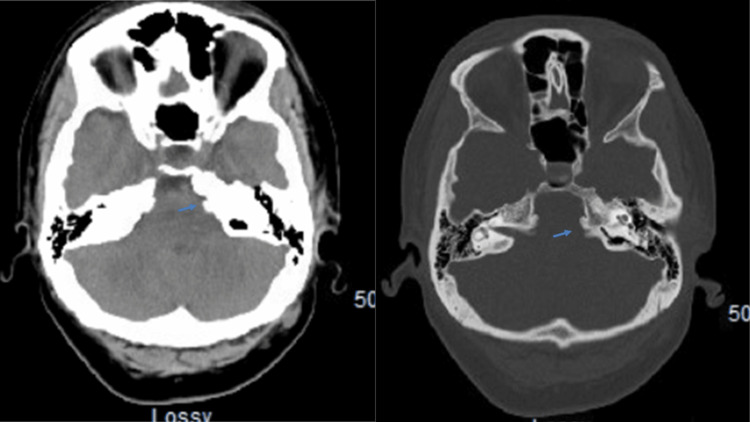
Preoperative axial head CT The view shows a thickened left petrous bone (blue arrow) CT: computed tomography

**Figure 2:**
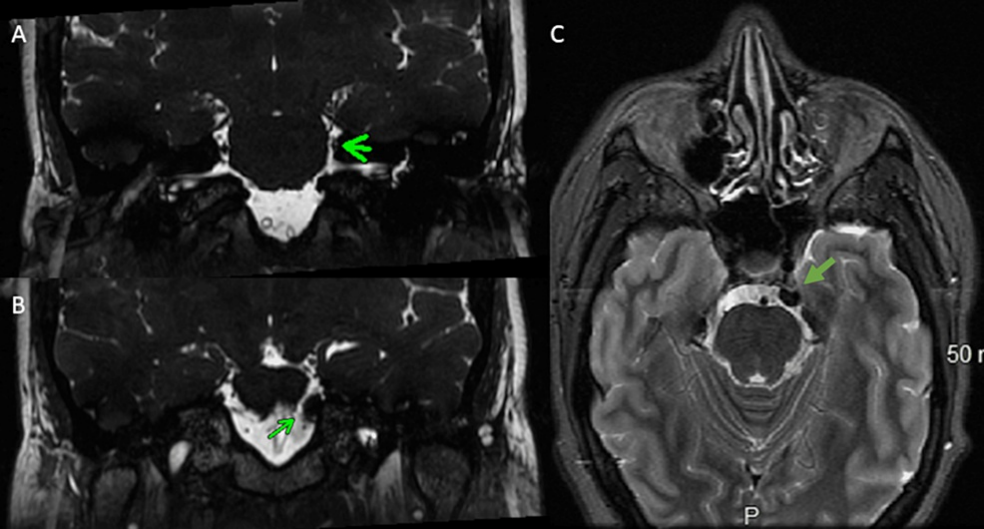
Preoperative brain MRI, coronal view (A and B) and axial view (C) The view shows osteomatous growths along the left petrous bone compressing the trigeminal nerve MRI: magnetic resonance imaging

Her physical examination was unremarkable except for left facial numbness. The patient was given a diagnosis of TN, and various pain medications, such as Tegretol, were tried for pain management. The results from medication were unsatisfactory, and after attempting other ways of treatment, she eventually opted for surgical intervention. Head computed tomography (CT) and magnetic resonance imaging (MRI) showed osteomatous growths along the left petrous bone that compressed the trigeminal nerve (Figures 1-2).

**Figure 3:**
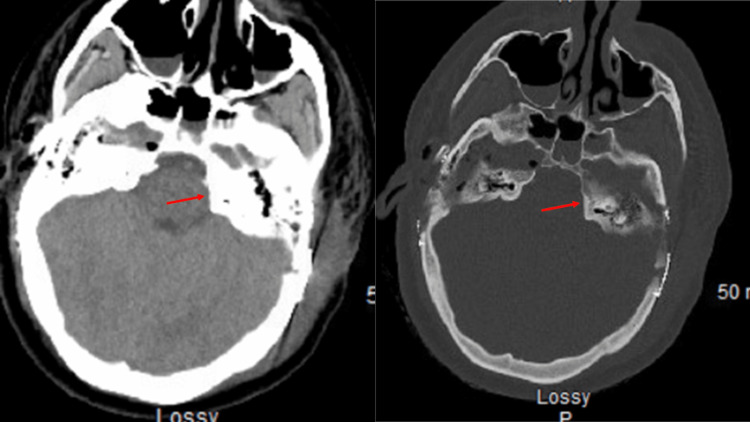
Postoperative axial head CT The view shows no compression on the trigeminal nerve after drilling out the protruding petrous bone CT: computed tomography

A retrosigmoid approach was discussed among other surgical options with the risks explained to the patient. A left retrosigmoid craniotomy was initially attempted but aborted due to intraoperative cerebral swelling. After a couple of days, a left middle fossa craniotomy was performed. The protruding petrous bone was drilled out to relieve trigeminal nerve compression. Postoperatively, head CT results showed no compression on the trigeminal nerve (Figure 3). The patient had no symptoms except for bilateral radiculopathy, which was worse when sitting down, and left-sided facial numbness and tingling. However, her symptoms resolved less than two months after the surgery.

**Figure 4:**
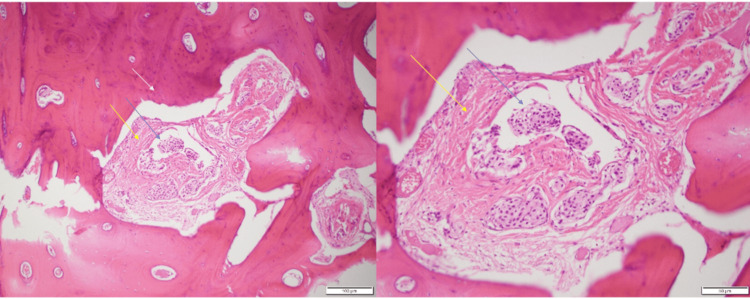
Microscopic examination of the resected bony lesion stained by hematoxylin and eosin The view shows fragments of reactive bone (white arrow) with nests of meningothelial cells (blue arrow) in intertrabecular spaces with whorl formation and collagen deposition (yellow arrow)

**Figure 5:**
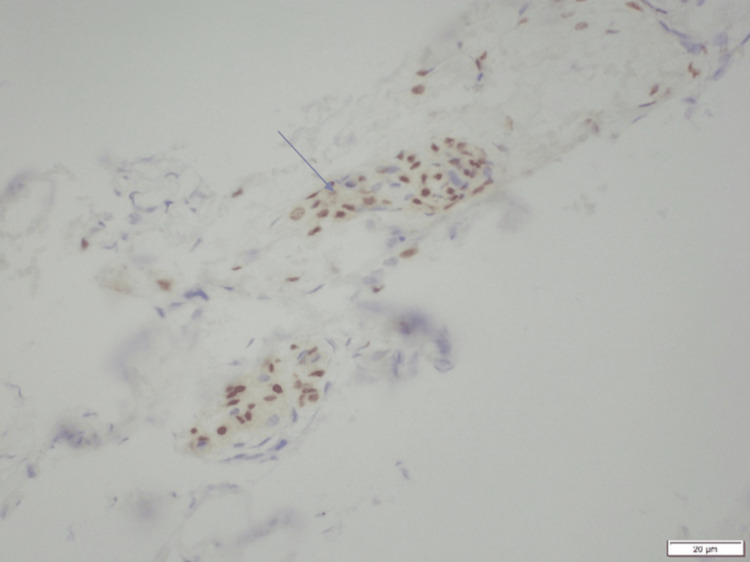
Microscopic examination of the resected bony lesion stained by progesterone immunostain The view shows progesterone-positive meningothelial cells

The microscopic examination of the resected bone lesion shows an intraosseous meningioma, WHO grade 1. The specimen showed fragments of reactive bone with nests of meningothelial cells in intertrabecular spaces with whorl formation and collagen deposition. Round to oval nuclei were observed with frequent intranuclear pseudoinclusions and syncytial arrangement of the cytoplasm. There were no definitive meningiomas observed outside bony fragments. There were no high-grade morphologic variants, high-grade features, necrosis, or mitotic figures (Figures 4-5). A two-month follow-up shows complete improvement in the patient’s symptoms.

## Discussion

Trigeminal nerve compression by mass lesions or blood vessels causes focal demyelination in the compressed region of the TN axon [1,2,8]. Demyelinated regions can be spontaneously active and produce ectopic impulses as demonstrated experimentally by Smith and McDonald [12] and Smith and Hall [13]. These ectopic impulses and the activity of the TN cause recurrent shock-like brief pain that can be evoked by many other stimuli, such as face-related tasks of daily hygiene or light touching [1,2,12,13]. This phenomenon can be explained by the ephaptic transmission of impulses between adjacent nonmyelinated nerves as demonstrated experimentally by Ramón and Moore [14].

The medical treatment of TN includes antiepileptics and tricyclic antidepressant medications. Carbamazepine is the first-line treatment for TN [2,15,16]. Surgical treatment for patients with non-remitting symptoms includes vascular decompression, mass decompression, and neuro-ablation via rhizotomy [2,16]. In our case, pharmacologic therapy was first attempted for treatment through carbamazepine and pregabalin without sufficient outcome, so a left middle fossa craniotomy was performed to decompress the trigeminal nerve by drilling out the protruding petrous bony structure.

Mechanical compression on the trigeminal nerve by petrous bony mass lesions is rare [8-11]. Our literature review showed that only four cases of trigeminal neuralgia caused by petrous bone growths are reported (Table 1) [8-11]. Wang et al. demonstrated the first reported case of trigeminal neuralgia caused by a petrous bone osteoma associated with ipsilateral clivus meningioma [9]. A subtemporal transtentorial approach was performed to resect the mass lesion [9]. Pathology identified the tumors to be an osteoma and meningioma, and the patient had decreased symptoms of trigeminal neuralgia over three months of follow-up [9]. Here, we highlight the importance of petrous bone lesions as a cause of TN.

**Table 1:** Reported cases of petrous bone growths causing trigeminal neuralgia in the literature

Reference	First author	Location	Association with meningioma	Presenting symptoms	Number of cases
[9]	Liang Wang	Petrous bone	Yes	Trigeminal neuralgia	One
[10]	Antonio Ruelle	Petrous bone	No	Trigeminal neuralgia	Two
[11]	Walter E. Dandy	Petrous bone	No	Trigeminal neuralgia	One
Our case	Phoebe Lim	Petrous bone	Yes	Trigeminal neuralgia	One

## Conclusions

TN may arise from a series of different nerve and vascular concerns, and the compression of the fifth cranial nerve is known as a common cause of TN. To our knowledge, this is the fifth reported case of TN resulting from petrous bony structure abnormalities in the current literature. Our patient presents with TN caused by the growth of an intraosseous meningioma along the left petrous bone, resulting in trigeminal nerve compression. The patient underwent petrous bone decompression through a middle cranial craniotomy. Postoperatively and at two-month follow-up, the patient’s pain was completely resolved. This case calls attention to trigeminal neuralgia as a consequence of petrous bone lesions.
